# Dietary Cholesterol Intake and Sources among U.S Adults: Results from National Health and Nutrition Examination Surveys (NHANES), 2001–2014

**DOI:** 10.3390/nu10060771

**Published:** 2018-06-14

**Authors:** Zhe Xu, Scott T. McClure, Lawrence J. Appel

**Affiliations:** 1 Welch Center for Prevention, Epidemiology, and Clinical Research, Johns Hopkins Bloomberg School of Public Health, Baltimore, MD 21287, USA; zxu43@jhu.edu (Z.X.); smcclur7@jhu.edu (S.T.M.); 2 Department of Epidemiology, Johns Hopkins Bloomberg School of Public Health, Baltimore, MD 21287, USA; 3 Division of General Internal Medicine, Johns Hopkins University, Baltimore, MD 21287, USA

**Keywords:** diet, cholesterol, NHANES-WWEIA, food groups

## Abstract

The 2015 Dietary Guidelines for Americans recommends that individuals should minimize their dietary cholesterol intake. However, current dietary cholesterol intake and its food sources have not been well-characterized. We examined dietary cholesterol intake by age, sex, race, and food sources using 24-h dietary recall data from a nationally representative sample of 5047 adults aged 20 years or older who participated in NHANES (2013–2014 survey cycle). We also reported trends in cholesterol intake across the past seven NHANES surveys. Mean dietary cholesterol intake was 293 mg/day (348 mg/day for men and 242 mg/day for women) in the 2013–2014 survey cycle; 39% of adults had dietary cholesterol intake above 300 mg/day (46% for men and 28% for women). Meat, eggs, grain products, and milk were the highest four food sources of cholesterol, contributing to 96% of the total consumption. Both average cholesterol intake and food source varied by age, sex, and race (each *p* < 0.05). Mean cholesterol intake of the overall population had been relatively constant at ~290 mg/day from 2001–2002 to 2013–2014 (*p*-trend = 0.98). These results should inform public health efforts in implementing dietary guidelines and tailoring dietary recommendations.

## 1. Introduction

A high intake of dietary cholesterol raises blood cholesterol levels, which is a major risk factor for cardiovascular disease [1]. In addition, a high intake of cholesterol has been associated with an increased risk of type 2 diabetes [2], liver disease progression [3], and several types of cancer [4]. While the direct effect of dietary cholesterol intake on cardiovascular disease risk in the general population is actively debated, the direct relationship of dietary cholesterol with cardiovascular disease in patients with diabetes is well-accepted [5].

In 2010, the 7th Dietary Guidelines for Americans recommended no more than 300 mg/day of cholesterol for healthy populations in the United States [6]. In 2015, Dietary Guidelines removed this recommendation [7]. However, the guidelines simultaneously stated that this change does not indicate that dietary cholesterol is no longer important to be considered, and that “individuals should eat as little dietary cholesterol as possible while consuming a healthy eating pattern”.

To our knowledge, the contribution of different food groups to total cholesterol intake in the general U.S. population (overall and among key age, sex, and race groups) has not been well described. While all cholesterol originates from animal sources (meat, milk, and eggs), many foods not generally considered animal products (such as baked goods) are made with cholesterol-containing ingredients. Such information would be important for implementing the guidelines and informing dietary interventions for disease prevention.

In order to estimate total and source-specific, dietary cholesterol intake among the U.S. adult population, we analyzed the data on food sources from National Health and Nutrition Examination Survey (NHANES)—What We Eat in America (WWEIA) 2013–2014 cycle. We also described differences in total and source-specific cholesterol intake by age, sex, and race groups and compared demographic and dietary patterns by level of total dietary cholesterol intake. In addition, we documented trends in total cholesterol intake across seven NHANES-WWEIA cycles from 2001–2014.

## 2. Materials and Methods

The National Health and Nutrition Examination Survey (NHANES) is an ongoing program of studies designed to assess the health and nutritional status of the civilian noninstitutionalized populations of the United States [8]. The NHANES were conducted by the National Center for Health Statistics (NCHS) of the Centers for Disease Control (CDC). Approximately 5000 nationally representative individuals are sampled every year by complex, multistage, stratified, clustered sampling method [8]. The NHANES protocol was reviewed and approved by the NCHS research ethics board. All participants provided written informed consent before participation.

In NHANES, dietary intake information was collected by 24-h dietary recall interviews from What We Eat in America (WWEIA) survey, to estimate the types and amounts of foods and beverages consumed during the 24-h period prior to the interview, as well as to estimate intakes of energy, nutrients, and other food components from those foods and beverages [9]. Cholesterol and energy intake were assigned to foods based on the nutrient database of the U.S. Department of Agriculture (USDA) [10].

We used data of NHANES from the 2013–2014 survey cycle, which was the latest released dataset with dietary measurements, to estimate the mean dietary cholesterol intake level among U.S. adults. Two dietary recall interviews were conducted during the survey. The first recall was administered in person, and the second recall was administered three to ten days later by telephone interview. We also examined trends of cholesterol intake of from 2001–2002 survey cycle to 2013–2014 survey cycle. However, before 2003–2004 NHANES survey cycle, dietary data was obtained only by using a single 24-h recall [11,12]. In our analyses, we only used the first dietary recall in order to maintain consistency across survey cycles and to maximize sample size, because some participants did not complete both diet recalls in the last cycle.

There were 10175 individual records in the 2013–2014 dataset. After excluding participants under age 20 (*n* = 4406) and those with incomplete dietary records (*n* = 722), we included 5047 adult men and women (20 years of age or older) with complete first 24-h dietary interviews in our analysis. Based on the same eligibility criteria, we included 34,741 participants from seven NHANES survey cycles between 2001 and 2014 for trend analyses.

We assessed the mean and 95% confident intervals (CIs) for dietary cholesterol intake (in mg/day) and energy intake (in kcal/day), as well as cholesterol density (in mg/1000 kcal), which was defined as the ratio of cholesterol intake to energy intake. We conducted stratified analyses among the study population based on sex, age (20–29, 30–49, 50–69, and 70+), and race (Non-Hispanic white, Non-Hispanic black, Mexican American, and other). Wald tests were used to test for statistically significant differences between the dietary intake estimates across different demographic characteristics within each subgroup. To better understand trends of mean cholesterol intake over the past more than 10 years, we used data from seven NHANES survey cycles, 2001–2002 to 2013–2014. We calculated age and sex adjusted means of the seven survey cycles separately by direct standardization to the 2000 U.S. Census population [13]. Trends across survey cycle were tested based on a weighted linear regression in which we modeled survey cycle as an ordinal variable and adjusted for age, sex, and race. Subgroup estimates of cholesterol intake from 2001–2002 to 2013–2014 were also conducted by sex, age, and race.

Dietary cholesterol intake from different food sources were estimated. The food group classification was based on the USDA nine food groups definition: milk and milk products (milk); meat, poultry, fish and mixtures (meat); eggs; legumes, nuts and seeds (nuts); grain products (grains); fruits; vegetables; fats, oils and salad dressings (fats); and sugar, sweeteners and beverages (beverages) [14]. We divided meats into red meat (beef, pork, ham, liver, and other organ meats), poultry (chicken, turkey), processed meat (bacon, sausage, other processed meats), seafood (fish, shellfish), and mixed dishes [15] in order to better understand the main sources of dietary cholesterol. We also divided grain products into cooked grains/cereals, breads/breads products, ready-to-eat (RTE) cereals, and other grain products (not cooked cereals, grain mixtures, and meat substitutes) [10,16] to characterize the details of cholesterol intake from different types of grain products. Finally, we compared the demographic and cholesterol intake from different food sources by sex-specific quartile of total dietary cholesterol intake. We compared the amount of individual food items intake and other nutrients intake across the quartiles. P-values for trends of cholesterol quartiles were tested using weighted linear regressions, modeling cholesterol quartiles as an ordinal variable.

All analyses accounted for the complex survey sample design of NHANES to produce national, population-based estimates of dietary cholesterol intake. Estimated mean total cholesterol intake was calculated using the svy: mean command, individual food files, and dietary one-day sample weights. Estimated percentages of dietary cholesterol intake coming from different food groups were calculated using the svy: proportion command, the individual food files, and dietary one-day sample weights. All analyses were performed with Stata statistical software version 14 (StataCorp LP, College Station, TX, USA) under “*svy*” command with probability weight (*pweight*).

## 3. Results

### 3.1. Overall Dietary Cholesterol Intake

**Figure 1:**
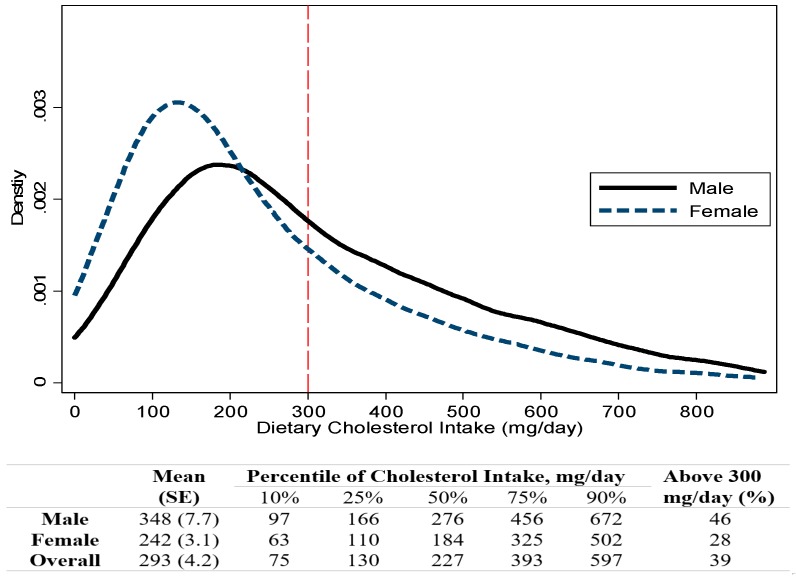
Histogram ^1^ of estimated cholesterol intake by gender for the U.S. adults 20 years of age or older, NHANES 2013–2014. ^1^ Truncated at 97.5 percentile of cholesterol intake.

Based on 2013 to 2014 NHANES survey cycle data, the estimated national population-level mean dietary cholesterol intake among adults 20 years of age or older was 293 mg/day (95% CI, 284–302), and the mean cholesterol density was 137 mg cholesterol per 1000 kcal energy intake (95% CI, 133–141) (Table 1). In 2013–2014, a total of 39% adults had dietary cholesterol intake above 300 mg/day (46% for men and 28% for women) (Figure 1). Compared to women, men had significantly higher total cholesterol intake (*p* <0.001) and higher cholesterol density (*p* = 0.031). A trend of decreased cholesterol intake with increased age was observed (*p*-trend = 0.001), but cholesterol density did not differ by age groups (*p*-trend = 0.48). Among the race-ethnicity groups, Mexican Americans had the highest cholesterol intake and highest cholesterol density, while Non-Hispanic whites consumed the least amount of cholesterol and had the lowest cholesterol density (*p* = 0.003 for cholesterol intake, *p* = 0.004 for cholesterol density comparison, respectively).

### 3.2. Trends of Dietary Cholesterol Intake

The age and sex adjusted mean total cholesterol intake from 2001 to 2014 of the overall population was 288 mg/day (95% CI, 285–292) (Table S1, Figure S1). There was no statistically significant change in both crude and adjusted total cholesterol intake from 2001–2002 to 2013–2014 (*p*-trend = 0.90 for crude mean intake and 0.98 for adjusted mean intake). In addition, cholesterol density remained stable from 2001 to 2014 (*p*-trend = 0.34 for adjusted values).

Although the total cholesterol intake varied among different subgroup populations by sex, age, and race, the patterns of dietary intake trends were similar for all subgroups: no significant changes of cholesterol intake over the past 12 years (each *p*-trend value >0.05) (Table S2).

### 3.3. Food Source for Cholesterol Intake

Among the overall population, meat, eggs, grain products, and milk contributed to 96% of the total dietary cholesterol consumption (Table 2). Meat contributed 42% to the total cholesterol intake (12% for poultry, 12% for mixed dishes, 8% for red meat, 5% for processed meat, and 5% for seafood) (Table S3), with 25% from eggs, 17% from grain products (5% for breads, 0% for cooked grains/cereals and RTE cereals, and 12% for other grain products), and 11% from milk and milk products.

**Figure 2:**
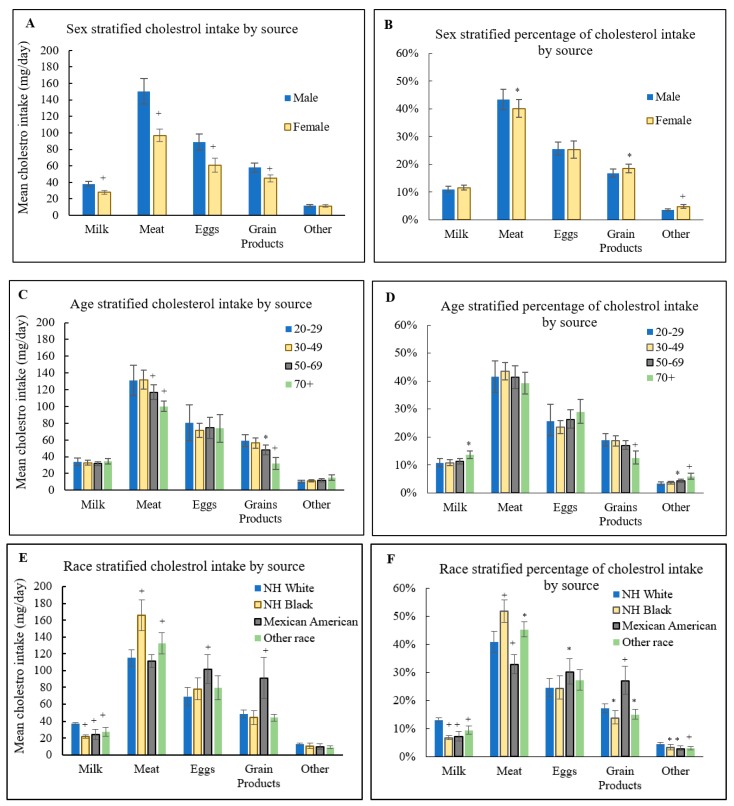
Mean Dietary Cholesterol Intake (95% confidence intervals) (**A**,**C**,**E**) and Percentage (95% confidence intervals) (**B**,**D**,**F**) by Age, Sex, and Race for the U.S. adults 20 years of age or older, NHANES, 2013–2014. * *p* < 0.05, + *p* < 0.01.

Figure 2 displays sex, age, and race stratified, mean cholesterol intake by source (left panels A, C, E) and corresponding percentages (right panels B, D, F). Men were more likely to consume more cholesterol from milk, meat, eggs, and grain products than women (panel A, each *p* < 0.001), but the percentages of total cholesterol intake from milk and eggs were similar for men and women (panel B, each *p* > 0.05). Cholesterol intake from milk and eggs was similar across age groups (panel C, each *p* > 0.05). However, people aged 50–69 and over 70 years were more likely to have less absolute cholesterol intake from meat and grain products compared to adults aged 20–29 years (panel C, each *p* < 0.05 for meat/grain products comparison), but for the percentage of cholesterol intake, only milk and grain products percentage differed compared over 70 aged adults to 20–29 aged adults (panel D, *p* = 0.029, *p* = 0.001 for milk and grain products comparison, respectively). Compared to Non-Hispanic whites, both Non-Hispanic blacks and Mexican Americans had lower cholesterol intake from milk (each *p* < 0.001); Mexican Americans had higher total cholesterol intake from eggs (panel E, *p* = 0.003) and grain products (panel E, *p* = 0.002), while Non-Hispanic blacks had higher total cholesterol intake from meat (panel E, *p* < 0.001). For the percentage of cholesterol intake, both Non-Hispanic blacks and Mexican Americans had lower proportion from milk compared to Non-Hispanic whites (panel F, each *p* < 0.001); Mexican Americans had a larger proportion from eggs (panel F, *p* = 0.047) and grain products (panel F, *p* = 0.002), but a lower proportion from meat (panel F, *p* = 0.007) than Non-Hispanic whites; Non-Hispanic blacks had a larger proportion from meat (panel F, *p* <0.001) but a lower proportion from grain products (panel F, *p* = 0.025) than Non-Hispanic whites.

Within meat group, women had a lower proportion of cholesterol intake from red meat and processed meat compared to men (Table S3). Compared to Non-Hispanic whites, Non-Hispanic blacks had a higher proportion of cholesterol intake from poultry and seafood; while Mexican Americans had a lower proportion of cholesterol intake from processed meat.

### 3.4. Factors Related to High Cholesterol Intake

Table 3 and Table 4 display demographic characteristics, food intake, and nutrient intake by quartiles of total cholesterol intake, separately for men and women. Among men, those with higher cholesterol intake had higher energy intake and cholesterol density, and were more likely to be Mexican Americans. Similar patterns were observed among women, but there was no significant difference by race-ethnicity for women (*p* = 0.31).

Cholesterol intake from different sources varied greatly across the quartiles, especially for eggs (1 mg/day for quartile 1 vs. 307 mg/day for quartile 4 among men). While meat was the primary source in quartiles 1 to 3, eggs were the predominant source in quartile 4. Among women, the findings were similar, but the magnitude of differences across quartiles were not as great as that observed in men.

Among men, those with higher cholesterol intake also had higher amount of daily food intake of milk, meat, eggs, grain products, vegetables, and fats/oils (Table S4). Among women, similar trends were observed (Table S5). For both men and women, those who had higher cholesterol intake also had higher intake of most nutrients than other participants, especially for the intake of protein, carbohydrate, total sugars, total fat, total saturated fatty acids, total monounsaturated fatty acids, total polyunsaturated fatty acids, vitamin C, calcium, and sodium.

## 4. Discussion

In our analyses of NHANES, a nationally representative survey, we documented that mean dietary cholesterol intake was 293 mg/day (348 mg/day for men and 242 mg/day for women) in 2013–2014. Accordingly, nearly half of men had daily cholesterol consumption over 300 mg. Mean total cholesterol intake was greater than 300 mg/day among young adults (20–29 years old), non-Hispanic blacks, and Mexican-Americans.

Compared with previous NHANES study cycles, the mean cholesterol intake among U.S. adults in 2013–2014 was roughly the same as that in 2005–2006, and 2007–2008 (around 290 mg/day), revealing a relative stable average cholesterol consumption in recent years. Compared with other countries, the current estimate was higher than the global level of mean cholesterol intake (228 mg/day) in 2010 [17], higher than some countries in South Asia and Africa, similar to some European countries (e.g., U.K. and Italy), but lower than some other European countries (e.g., Romania and Denmark) and developed Asian countries (e.g., Japan).

In our analyses, meat was the largest contributor of dietary cholesterol; however, among those with the highest intake of dietary cholesterol, eggs were the primary source. People with higher cholesterol intake also tended to have higher energy intake and higher consumption of most foods and nutrients. A meta-analysis of 22 cohorts had concluded that high egg consumption was associated with an increased type 2 diabetes risk among some of the general population and cardiovascular disease comorbidity among diabetic patients [5]. For people with extremely high dietary cholesterol intake, especially for diabetic patients or those with high risk of cardiovascular disease, reducing egg intake should lower excessive cholesterol intake effectively. Grain products also contributed a large percentage of total cholesterol intake. Even though many grain products are cholesterol free, certain grain products such as biscuits, egg pasta, and mixed dishes provide dietary cholesterol.

Among meat groups, red/processed meat, mixed dishes, and poultry also contribute large amounts of food cholesterol intake. These findings were in line with the American Heart Association (AHA) recommendations that when choosing a meat product, people are encouraged to select from seafood/skinless poultry (but less poultry organ meats) and trimmed lean meat, combining that with healthier cooking methods [18] to avoid excessive cholesterol intake from meat. The food sources of cholesterol intake also varied among different populations: men, younger and non-Hispanic Black population had higher cholesterol intake from meat, while Mexican Americans had more cholesterol intake from egg and grain products. These differences suggest that health education and intervention programs regarding dietary quality improvement should be more nuanced and tailored for diverse populations.

Our study has several strengths. First, we estimated current and prior dietary cholesterol intake from surveys of nationally representative U.S. adults. The surveys were sufficiently large to provide subgroup estimates by age, sex, and race, and to test trends. Second, robust data on food sources allowed us to identify broad food sources of dietary cholesterol intake, as well as meat-specific sources. The study also has certain limitations intrinsic to diet assessment. Diet was measured using self-reported 24-h dietary recalls; such instruments are potentially affected by recall bias, but provide more detailed intake data than other diet measurement tools, such as food frequency questionnaires. Only the first day of dietary data was used in our study in order to maintain consistency across survey cycles and to maximize sample size. A single 24-h dietary recall may not accurately reflect an individual’s usual diet; however, such results provide reasonable estimates of actual population means [19,20].

Our study has policy implications. Previously, the Dietary Guidelines for Americans recommended that individuals limit consumption of dietary cholesterol to 300 mg/day [6]. But this recommendation was not brought forward to the 2015 Dietary Guidelines. The 2015 guidelines noted that people do not need to obtain cholesterol through foods because the body can make more than enough cholesterol for normal functions. Still, the 2015 Dietary Guidelines recommend that individuals should eat as little dietary cholesterol as possible for a healthy eating pattern [7]. Variation in amount and source of dietary cholesterol by age, gender, and race, as reported in this paper, could help guide public health efforts in tailoring dietary recommendations. For example, in Mexican-Americans, a larger amount and percentage of dietary cholesterol came from grain products; in non-Hispanic blacks and in men, a larger amount and percentage of cholesterol came from meats.

## 5. Conclusions

Mean cholesterol intake among U.S. adults in 2013–2014 was approximately 290 mg/day and has remained unchanged over the past 10 years. Still, 46% of male and 28% of female adults had daily cholesterol consumption over 300 mg. Both average cholesterol intake and source of dietary cholesterol varied by age, sex, and race. While meat, overall, is the primary dietary source of cholesterol; eggs are the predominant source among those with high intake of cholesterol. These results should inform public health efforts in implementing dietary guidelines and tailoring dietary recommendations.

## Figures and Tables

**Table 1:** Estimated means (95% confidence intervals) for total dietary cholesterol intake, energy intake, and cholesterol density for the U.S. adults 20 years of age or older, NHANES 2013–2014.

	*N* (Unweighted)	Mean Cholesterol Intake (mg/day)	Mean Energy Intake (kcal/day)	Cholesterol Density (mg/1000 kcal)
Overall	5047	293 (284–302)	2141 (2101–2181)	137 (133–141)
Sex				
Male (ref.)	2414	348 (331–364)	2477 (2422–2533)	144 (138–150)
Female	2633	242 (235–249) ^+^	1825 (1787–1864) ^+^	135 (130–140) *
Age group				
20–29 (ref.)	854	315 (293–337)	2328 (2210–2445)	139 (127–152)
30–49	1789	303 (290–317)	2245 (2192–2297)	137 (132–0.142)
50–69	1665	283 (268–299) *	2054 (1979–2130) ^+^	139 (135–144)
70+	739	255 (232–279) ^+^	1789 (1717–1861) ^+^	146 (137–155)
Race				
Non-Hispanic White (ref.)	2233	282 (273–291)	2129 (2080–2178)	135 (131–140)
Non-Hispanic Black	1009	320 (301–339) ^+^	2230 (2113–2347)	142 (137–147) *
Mexican American	669	338 (304–372) ^+^	2256 (2131–2381)	153 (143–163) ^+^
Other	1136	292 (268–316)	2050 (1957–2142)	146 (137–156) *

* *p* < 0.05, ^+^ *p* < 0.01 for comparison.

**Table 2:** Estimated total dietary cholesterol intake (mg/day) and percentage (%) by nine USDA food groups for US adults aged ≥20 years in NHANES, 2013–2014.

	Milk ^a^	Meat ^b^	Eggs	Nuts	Grain Products	Fruits	Vegetables	Fats ^c^	Sugar ^d^	All
Overall	33 (11.2)	123 (41.9)	74 (25.4)	0 (0.1)	51 (17.5)	0 (0)	5 (1.6)	5 (1.6)	2 (0.6)	293 (100)
Sex										
Male (ref.)	38 (11.0)	151 (43.3)	89 (25.6)	0 (0.1)	58 (16.7)	0 (0)	5 (1.4)	5 (1.4)	2 (0.5)	348 (100)
Female	28 (11.6)	97 (40.1) *	61 (25.2)	0 (0.1)	45 (18.5)	0 (0)	5 (1.9)	4 (1.8) ^+^	2 (0.8) *	242 (100)
Age group										
20–29 (ref.)	34 (10.7)	131 (41.5)	81 (25.6)	0 (0.1)	59 (18.9)	0 (0)	4 (1.3)	4 (1.3)	2 (0.7)	315 (100)
30–49	33 (10.8)	132 (43.5) *	71 (23.5)	0 (0.1)	56 (18.6) *	0 (0)	4 (1.4)	4 (1.3)	2 (0.8)	303 (100)
50–69	32 (11.2)	117 (41.3)	74 (26.3)	0 (0.1)	48 (17.0) ^+^	0 (0)	5 (1.8)	5 (1.7)	2 (0.6)	283 (100)
70+	35 (13.5) *	100 (39.2)	74 (28.9)	1 (0.2)	32 (12.5) ^+^	0 (0)	7 (2.7) ^+^	6 (2.4) ^+^	1 (0.4)	255 (100)
Race										
Non-Hispanic White (ref.)	37 (13.2)	115 (40.8)	69 (24.4)	0 (0.1)	48 (17.1)	0 (0)	5 (1.8)	5 (1.8)	2 (0.7)	282 (100)
Non-Hispanic Black	22 (6.7) ^+^	166 (51.8) ^+^	78 (24.4)	0 (0.1)	44 (13.8)	0 (0)	4 (1.4)	4 (1.3) *	1 (0.4)	320 (100)
Mexican American	24 (7.2) ^+^	111 (32.8) *	102 (30.1) ^+^	0 (0.1)	91 (27.0) ^+^	0 (0)	5 (1.4)	3 (0.9) ^+^	1 (0.4)	338 (100)
Other	27 (9.4) ^+^	132 (45.3) *	79 (27.2)	0 (0.2)	44 (15.1)	0 (0)	3 (1.1) *	3 (1.1) ^+^	2 (0.7)	292 (100)

^a^ Milk refers to Milk and Milk Products. ^b^ Meat refers to Red Meat, Poultry, Processed Meat, Seafood and Mixed Dishes. ^c^ Fats, Oils & Salad Dressings. ^d^ Sugar, Sweeteners & Beverages. * *p* < 0.05, ^+^ *p* < 0.01 for percentage of dietary cholesterol intake comparison.

**Table 3:** Characteristics of one-day dietary measures by dietary cholesterol intake quartiles for U.S. men aged ≥20 years in NHANES, 2013–2014.

	Overall	Q1	Q2	Q3	Q4	*p*-Trend *
*N* (unweighted)	2414	612	596	603	603	
Cholesterol intake, mean(SE), mg/day	348 (7.7)	101 (2.1)	216 (1.4)	368 (3.2)	731 (18.1)	
Cholesterol intake, range, mg/day	(0, 2584)	(0, 160)	(161, 277)	(278, 476)	(477, 2584)	
Total energy intake, mean (SE), kcal/day	2477 (26.1)	1793 (38.7)	2325 (4.6)	2643 (40.5)	3176 (73.4)	<0.001
Cholesterol density, mean (SE), mg/1000 kcal	144 (2.8)	64 (1.7)	104 (2.1)	156 (2.3)	260 (6.0)	<0.001
Age, mean (SE), years	47 (0.4)	49 (1.0)	47 (0.7)	46 (0.8)	46 (1.0)	0.028
Race, %						
Non-Hispanic White	66	65	73	66	59	0.006
Non-Hispanic Black	11	12	9	8	14	
Mexican American	10	8	6	13	12	
Other race	14	15	12	13	15	
Cholesterol intake by source, mean (SE), mg/day						
Milk and milk products	38 (1.4)	20 (1.0)	36 (2.4)	49 (3.7)	47 (3.2)	0.006
Eggs	89 (4.5)	1 (0.7)	9 (1.9)	58 (5.6)	307 (15.9)	<0.001
Meat	151 (7.3)	50 (1.9)	116 (3.9)	181 (9.2)	260 (17.0)	<0.001
Red meat	32 (3.1)	10 (0.9)	28 (2.4)	35 (3.9)	59 (10.5)	<0.001
Poultry	42 (4.1)	11 (1.2)	26 (3.6)	50 (6.9)	86 (10.7)	<0.001
Processed meat	19 (1.3)	10 (0.9)	16 (1.6)	24 (2.2)	28 (3.9)	<0.001
Seafood	17 (2.8)	5 (0.8)	9 (2.5)	19 (3.8)	37 (8.9)	0.002
Mixed dishes	40 (1.7)	14 (1.4)	36 (3.2)	55 (3.7)	55 (5.3)	<0.001
Grain products	58 (2.6)	24 (1.8)	44 (3.2)	69 (4.6)	98 (9.1)	<0.001
Others	12 (0.6)	7 (0.7)	11 (0.8)	12 (0.9)	19 (1.8)	<0.001

* *p*-trend values were calculated from a survey weighted linear regression modeling cholesterol intake quartiles as ordinal variable.

**Table 4:** Characteristics of one-day dietary measures by dietary cholesterol intake quartiles for U.S. women aged ≥20 years in National Health and Nutrition Examination Survey, 2013–2014.

	Overall	Q1	Q2	Q3	Q4	*p*-Trend *
*N* (unweighted)	2633	660	659	656	658	
Cholesterol intake, mean (SE), mg/day	242 (3.1)	69 (1.3)	149 (1.2)	255 (1.7)	519 (5.7)	
Cholesterol intake, range, mg/day	(0, 1944)	(0, 111)	(112, 191)	(192, 333)	(334, 1944)	
Total energy intake, mean (SE), kcal/day	1825 (18.1)	1332 (38.7)	1708 (31.4)	2047 (20.5)	2263 (66.6)	<0.001
Cholesterol density, mean (SE), mg/1000 kcal	135 (2.3)	59 (1.7)	98 (2.2)	139 (1.8)	254 (7.2)	<0.001
Age, mean (SE), years	48 (0.4)	49 (0.7)	48 (0.7)	48 (0.9)	47 (1.1)	0.13
Race, %						
Non-Hispanic White	65	66	68	67	61	0.32
Non-Hispanic Black	12	12	11	11	15	
Mexican American	9	8	8	8	10	
Other race	14	14	13	14	15	
Cholesterol intake by source, mean (SE), mg/day						
Milk and milk products	28 (1.0)	15 (1.2)	24 (0.9)	37 (2.4)	36 (4.0)	<0.001
Eggs	61 (4.0)	0 (0.1)	3 (0.7)	31 (3.5)	223 (11.8)	<0.001
Meat	97 (3.5)	32 (1.4)	73 (1.5)	117 (4.8)	173 (11.5)	<0.001
Red meat	17 (1.0)	5 (0.6)	13 (1.2)	21 (2.5)	30 (2.8)	<0.001
Poultry	27 (1.7)	6 (0.6)	21 (1.9)	32 (2.1)	49 (5.9)	<0.001
Processed meat	11 (0.7)	5 (0.6)	11 (1.8)	12 (1.3)	16 (1.7)	<0.001
Seafood	12 (1.9)	3 (0.6)	5 (1.2)	11 (2.3)	31 (5.7)	<0.001
Mixed dishes	32 (1.5)	11 (1.3)	22 (1.5)	43 (4.2)	53 (5.1)	<0.001
Grain products	45 (1.9)	15 (1.0)	39 (1.5)	56 (3.9)	71 (6.3)	<0.001
Others	11 (0.8)	7 (0.7)	9 (0.9)	14 (1.3)	16 (1.7)	<0.001

* *p*-trend values were calculated from a survey weighted linear regression modeling cholesterol intake quartiles as ordinal variable.

## Supplementary Materials

The following are available online at http://www.mdpi.com/2072-6643/10/6/771/s1, Figure S1: Estimated mean1 (95% confidence intervals) of total dietary cholesterol intake for the U.S. adults 20 years of age or older, in NHANES survey cycles: 2001–2002 to 2013–2014; Table S1: Crude and adjusted mean dietary cholesterol intake and cholesterol density (proportion of cholesterol of total calories) for the U.S. adults 20 years of age or older, NHANES survey cycles: 2001–2002 to 2013–2014; Table S2: Estimated means of total dietary cholesterol intake for the U.S. adults 20 years of age or older, NHANES survey cycles: 2001–2002 to 2013–2014; Table S3: Estimated total dietary cholesterol intake (mg/day) and percentage (%) by food groups with meat/grain products subgroups for US adults aged ≥20 years in NHANES, 2013–2014; Table S4: Food and nutrients intake of one-day dietary measures by dietary cholesterol intake quartiles for U.S. men aged ≥20 years in NHANES, 2013–2014; Table S5: Food and nutrients intake of one-day dietary measures by dietary cholesterol intake quartiles for U.S. women aged ≥20 years in NHANES, 2013–2014.
