# Supplementary material for: Dietary Cholesterol Intake and Sources among U.S Adults: Results from National Health and Nutrition Examination Surveys (NHANES), 2001–2014
Source: Nutrients. 2018 Jun 14;10(6):771. doi: 10.3390/nu10060771 (PMC6024549; doi:10.3390/nu10060771)
Supplement: Supplementary file 1 [file nutrients-10-00771-s001.pdf]

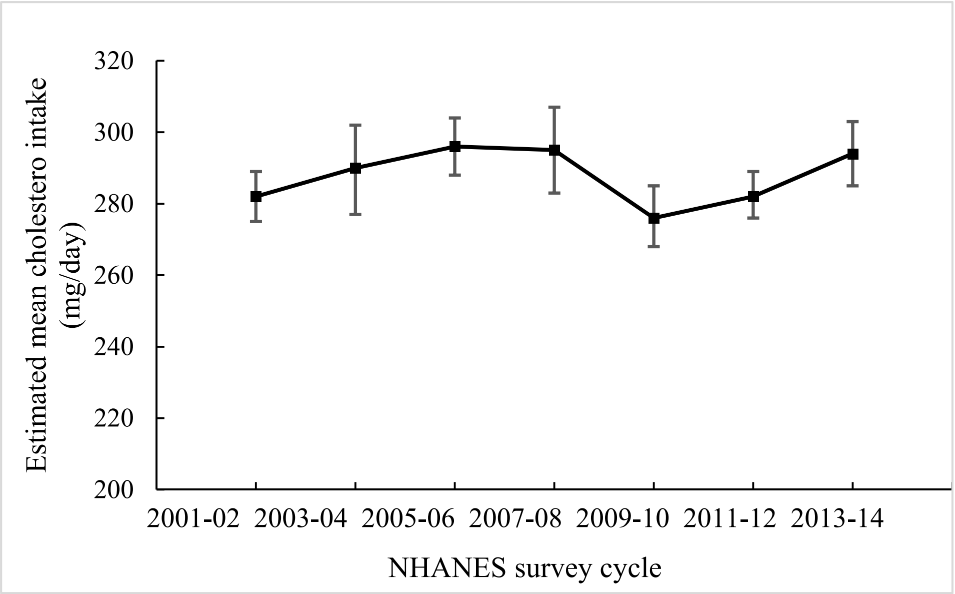

Figure S1. Estimated mean[1] (95% confidence intervals) of total dietary cholesterol intake for the U.S. adults 20 years of age or older, in NHANES survey cycles: 2001-2002 to 2013-2014

[1] Age and sex adjusted means of cholesterol intake by direct standardization to the 2000 US Census population

Table S1. Crude and adjusted* mean dietary cholesterol intake and cholesterol density (proportion of cholesterol of total calories) for the U.S. adults 20 years of age or older, NHANES survey cycles: 2001-2002 to 2013-2014

NHANES survey cycle	Mean intake (mg/day)		Density (mg/1000 kcal)	
	Crude	Adjusted	Crude	Adjusted
**Overall**	287 (284-291)	288 (285-292)	134 (133-136)	134 (133-136)
**2001-2002**	283 (275-291)	282 (275-289)	130 (126-133)	130 (126-133)
**2003-2004**	289 (276-302)	290 (277-302)	135 (130-139)	134 (129-139)
**2005-2006**	295 (286-303)	296 (288-304)	136 (133-139)	136 (133-139)
**2007-2008**	292 (280-304)	295 (283-307)	138 (134-142)	139 (135-142)
**2009-2010**	276 (267-285)	276 (268-285)	130 (126-135)	130 (125-134)
**2011-2012**	282 (275-289)	282 (276-289)	132 (128-136)	131 (127-136)
**2013-2014**	293 (285-301)	294 (285-303)	139 (136-142)	139 (136-142)
**P-trend**	0.895	0.977+	0.072	0.340+

* Adjusted mean intake was calculated by direct standardization to the 2000 US Census population, adjusted for age and sex.

+ Adjusted *p*-trend values were calculated from a survey weighted linear regression modeling survey cycle as ordinal variable, adjusted for race, age, and sex.

Table S2. Estimated means[1] of total dietary cholesterol intake for the U.S. adults 20 years of age or older, NHANES survey cycles: 2001-2002 to 2013-2014

	Overall	2001-02	2003-04	2005-06	2007-08	2009-10	2011-12	2013-14	p-trend*
**N (unweighted)**	34,741	4,744	4,448	4,520	5,419	5,762	4,801	5,047	
**Overall**	288	282	290	296	295	276	282	294	0.98
**Sex**									
**Male**	348	339	349	361	364	334	340	350	0.91
**Female**	232	229	235	236	231	223	228	242	0.72
**Age group**									
**20-29**	295	293	303	290	286	286	290	315	0.49
**30-49**	306	306	305	322	322	287	296	304	0.052
**50-69**	281	264	285	284	288	279	279	284	0.39
**70+**	232	219	228	245	229	219	229	250	0.13
**Race**									
**Non-Hispanic White**	282	278	282	292	292	268	274	284	0.56
**Non-Hispanic Black**	310	304	307	304	311	316	306	323	0.13
**Mexican American**	319	322	331	313	312	297	320	330	0.73
**Other**	275	251	279	290	276	260	270	288	0.43

[1] Age and sex adjusted means of cholesterol intake by direct standardization to the 2000 US Census population

* *p*-trend values were calculated from a survey weighted linear regression modeling survey cycle as ordinal variable, adjusted for race, age, and sex.

Table S3. Estimated total dietary cholesterol intake (mg/day) and percentage (%) by food groups with meat/grain products subgroups for US adults aged ≥20 years in NHANES, 2013–2014.

	Milk [a]	Eggs	Meat						Grain products					Other	All
			All meat	Red meat	Poultry	Processed meat	Seafood	Mixed dishes	All grain products	Cooked grains [b]	Breads [c]	RTE cereals [d]	Other grain products [e]		
**Overall**	33 (11.2)	74 (25.4)	123 (41.9)	24 (8.3)	34 (11.7)	15 (5.1)	15 (5.0)	36 (12.2)	51 (17.5)	1 (0.0)	14 (4.8)	0 (0.0)	36 (12.3)	12 (4.0)	293 (100)
**Sex**															
**Male (ref.)**	38 (11.0)	89 (25.6)	151 (43.3)	32 (9.4)	42 (12.2)	19 (5.6)	17 (4.9)	40 (11.5)	58 (16.7)	1 (0.0)	15 (4.3)	0 (0.0)	43 (12.4)	12 (3.5)	348 (100)
**Female**	28 (11.6)$^{+}$	61 (25.2)	97 (40.1)*	17 (6.9)$^{+}$	27 (11.0)	11 (4.6)$^{+}$	12 (5.1)	32 (13.2)	45 (18.5)	1 (0.0)	14 (5.8)*	0 (0.0)	30 (12.4)	11 (4.7)$^{+}$	242 (100)
**Age group**															
**20-29 (ref.)**	34 (10.7)	81 (25.6)	131 (41.5)	28 (8.8)	42 (13.2)	15 (4.6)	13 (4.3)	37 (11.8)	59 (18.9)	0 (0.0)	12 (3.8)	0 (0.0)	47 (14.9)	10 (3.3)	315 (100)
**30-49**	33 (10.8)	71 (23.5)	132 (43.5)*	28 (9.2)	37 (12.3)	15 (5.1)	16 (5.3)	35 (11.7)	56 (18.6)*	1 (0.0)	13 (4.3)	0 (0.0)	43 (14.2)	11(3.6)	303 (100)
**50-69**	32 (11.2)	74 (26.3)	117 (41.3)	21 (7.3)	31 (11.0)	15 (5.4)	14 (5.1)	36 (12.7)	48 (17.0)$^{+}$	1 (0.0)	16 (5.7)$^{+}$	0 (0.0)	31 (11.0)	12 (4.2)	283 (100)
**70+**	35 (13.5)	74 (28.9)	100 (39.2)	19 (7.3)	22 (8.7)	14 (5.7)	13 (4.9)	34 (13.2)	32 (12.5)$^{+}$	1 (0.0)*	16 (6.3)$^{+}$	0 (0.0)	15 (5.9)$^{+}$	15 (5.8)$^{+}$	255 (100)
**Race**															
**Non-Hispanic White (ref.)**	37 (13.2)	69 (24.4)	115 (40.8)	23 (8.2)	29 (10.2)	17 (5.9)	13 (4.5)	35 (12.4)	48 (17.1)	1 (0.0)	15 (5.3)	0 (0.0)	33 (11.7)	13 (4.5)	282 (100)
**Non-Hispanic Black**	22 (6.7)$^{+}$	78 (24.4)	166 (51.8)$^{+}$	28 (8.7)	57 (17.8)$^{+}$	16 (5.1)	27 (8.3)$^{+}$	45 (14.1)	44 (13.8)	1 (0.0)	15 (4.7)	0 (0.0)	28 (8.8)	11 (3.3)$^{+}$	320 (100)
**Mexican American**	24 (7.2)$^{+}$	102 (30.1)$^{+}$	111 (32.8)*	25 (7.5)	33 (9.9)	9 (2.7)$^{+}$	9 (2.6)	31 (9.2)*	91 (27)$^{+}$	0 (0.0)	14 (4.1)	0 (0.0)	77 (22.8)$^{+}$	10 (2.9)	338 (100)
**Other**	27 (9.4)$^{+}$	79 (27.2)	132 (45.3)*	26 (8.9)	41 (14.2)$^{+}$	10 (3.4)$^{+}$	18 (6.1)$^{+}$	35 (12.0)	44 (15.1)	1 (0.0)	12 (4.1)	0 (0.0)	31 (10.6)	9 (3.1)	292 (100)

[a] Milk refers to milk and milk products
[b] Cooked grains refer to cooked grains and cereals
[c] Breads refer to breads and breads products
[d] RTE cereals refer to ready-to-eat cereals
[e] Other grain products refer to not cooked cereals, grain mixtures, and meat substitutes
* p <0.05, $^{+}$ p <0.01 for percentage of dietary cholesterol intake comparison

Table S4. Food and nutrients intake of one-day dietary measures by dietary cholesterol intake quartiles for U.S. **men** aged ≥20 years in NHANES, 2013–2014.

	Overall	Q1	Q2	Q3	Q4	P-trend*
**N (unweighted)**	2414	612	596	603	603	
**Cholesterol intake, mean(SE), mg/day**	348 (7.7)	101 (2.1)	216 (1.4)	368 (3.2)	731 (18.1)	
**Cholesterol intake, range, mg/day**	(0, 2584)	(0, 160)	(161, 277)	(278, 476)	(477, 2584)	
**Food intake, mean (SE), mg/day**						
**Milk and milk products**	226 (10.8)	175 (12.5)	223 (16.4)	251 (20.7)	253 (19.9)	<0.001
**Meat, Poultry, Fish & Mixtures**	248 (7.6)	113 (6.2)	226 (8.8)	293 (13.0)	365 (18.7)	<0.001
**Eggs**	31 (1.4)	2 (0.9)	7 (2.1)	20 (1.9)	103 (5.0)	<0.001
**Legumes, Nuts and Seeds**	36 (2.1)	45 (5.0)	29 (3.9)	32 (3.8)	39 (4.1)	0.53
**Grain products**	373 (7.4)	307 (10.9)	363 (18.3)	396 (9.8)	426 (14.7)	<0.001
**Fruits**	147 (6.6)	160 (14.8)	128 (10.9)	139 (11.1)	165 (9.5)	0.63
**Vegetables**	180 (9.2)	137 (6.4)	174 (11.6)	179 (16.0)	231 (18.9)	<0.001
**Fats, Oils & Salad Dressings**	13 (0.9)	8 (1.0)	14 (1.9)	14 (1.6)	15 (1.7)	0.003
**Nutrients intake, mean (SE)**						
**Protein, mean (SE), gm/day**	98 (1.25)	58 (1.9)	87 (1.3)	108 (2.1)	141 (3.3)	<0.001
**Carbohydrate, mean (SE), gm/day**	287 (3.2)	240 (5.1)	280 (7.0)	302 (7.9)	327 (8.8)	<0.001
**Dietary fiber, mean (SE), gm/day**	19 (0.4)	17 (0.6)	19 (0.5)	19 (0.7)	21 (0.9)	0.004
**Total fat, mean (SE), gm/day**	95 (1.3)	59 (1.3)	86 (2.1)	103 (2.1)	134 (3.1)	<0.001
**Total saturated fatty acids, mean (SE), gm/day**	31 (0.5)	18 (0.4)	28 (0.8)	35 (0.9)	44 (1.0)	<0.001
**Total monounsaturated fatty acids, mean (SE), gm/day**	33 (0.4)	21 (0.5)	30 (0.7)	35 (0.7)	48 (1.1)	<0.001
**Total polyunsaturated fatty acids, mean (SE), gm/day**	22 (0.5)	15 (0.5)	20 (0.7)	23 (0.7)	30 (1.2)	<0.001
**Vitamin C, mean (SE), mg/day**	83 (2.4)	71 (5.1)	79 (4.1)	80 (5.6)	105 (6.9)	0. 008
**Vitamin D, mean (SE), mcg/day**	6 (0.3)	3 (0.3)	5 (0.2)	6 (0.3)	8 (1.0)	<0.001
**Calcium, mean (SE), mg/day**	1086 (18.3)	759 (22.9)	1058 (28.3)	1169 (35.9)	1363 (33.8)	<0.001
**Phosphorus, mean (SE), mg/day**	1625 (17.9)	1096 (33.7)	1502 (28.4)	1753 (33.8)	2171 (48.4)	<0.001
**Sodium, mean (SE), mg/day**	4094 (53.3)	2694 (48.1)	3767 (76.0)	4496 (95.6)	5470 (123.8)	<0.001
**Potassium, mean (SE), mg/day**	3016 (36.0)	2317 (59.7)	2875 (64.4)	3126 (71.4)	3782 (64.1)	<0.001

* *p*-trend values were calculated from a survey weighted linear regression modeling cholesterol intake quartiles as ordinal variable

Table S5. Food and nutrients intake of one-day dietary measures by dietary cholesterol intake quartiles for U.S. **women** aged ≥20 years in NHANES, 2013–2014.

	Overall	Q1	Q2	Q3	Q4	P-trend*
**N (unweighted)**	2633	660	659	656	658	
**Cholesterol intake, mean(SE), mg/day**	242 (3.10)	69 (1.3)	149 (1.2)	255 (1.7)	519 (5.7)	
**Cholesterol intake, range, mg/day**	(0, 1944)	(0, 111)	(112, 191)	(192, 333)	(334, 1944)	
**Food intake, mean (SE), mg/day**						
**Milk and milk products**	172 (6.1)	139 (9.0)	171 (5.9)	197 (12.2)	184 (10.8)	0.002
**Meat, Poultry, Fish & Mixtures**	173 (5.3)	86 (6.0)	152 (8.6)	212 (10.2)	251 (15.1)	<0.001
**Eggs**	24 (1.5)	2 (0.7)	3 (0.7)	15 (1.6)	79 (4.9)	<0.001
**Legumes, Nuts and Seeds**	26 (1.2)	29 (2.5)	21 (3.1)	27 (2.8)	28 (3.0)	0.80
**Grain products**	284 (5.3)	222 (8.5)	300 (9.6)	315 (7.7)	302 (12.9)	<0.001
**Fruits**	141 (6.0)	136 (9.9)	143 (11.0)	139 (9.9)	147 (15.4)	0.65
**Vegetables**	167 (4.4)	141 (6.8)	165 (10.4)	165 (9.2)	200 (10.3)	0.001
**Fats, Oils & Salad Dressings**	11 (0.7)	9 (1.0)	10 (0.9)	14 (1.4)	13 (1.4)	0.002
**Nutrients intake, mean (SE)**						
**Protein, mean (SE), gm/day**	70 (0.8)	43 (1.1)	62 (1.1)	80 (0.8)	97 (2.6)	<0.001
**Carbohydrate, mean (SE), gm/day**	220 (3.0)	184 (5.1)	217 (4.4)	239 (3.8)	244 (9.3)	<0.001
**Dietary fiber, mean (SE), gm/day**	99 (1.7)	83 (2.8)	96 (2.5)	107 (2.3)	110 (5.4)	<0.001
**Total fat, mean (SE), gm/day**	15 (0.3)	14 (0.5)	15 (0.5)	16 (0.4)	16 (0.5)	<0.001
**Total saturated fatty acids, mean (SE), gm/day**	71 (0.7)	45 (1.6)	64 (1.8)	83 (2.1)	96 (2.9)	<0.001
**Total monounsaturated fatty acids, mean (SE), gm/day**	23 (0.2)	13 (0.4)	21 (0.5)	28 (0.8)	31 (1.0)	<0.001
**Total polyunsaturated fatty acids, mean (SE), gm/day**	25 (0.3)	16 (0.7)	21 (0.7)	28 (0.7)	34 (1.1)	0.007
**Vitamin C, mean (SE), mg/day**	17 (0.2)	12 (0.6)	15 (0.6)	19 (0.5)	22 (0.8)	<0.001
**Vitamin D, mean (SE), mcg/day**	75 (2.2)	66 (3.6)	78 (4.3)	70 (3.8)	86 (3.9)	<0.001
**Calcium, mean (SE), mg/day**	4 (0.1)	3 (0.2)	3 (0.2)	5 (0.3)	6 (0.3)	0.004
**Phosphorus, mean (SE), mg/day**	852 (10.9)	642 (22.7)	840 (20.1)	971 (22.9)	971 (34.1)	<0.001
**Sodium, mean (SE), mg/day**	1187 (12.2)	820 (21.7)	1101 (21.8)	1339 (12.9)	1526 (37.7)	<0.001
**Potassium, mean (SE), mg/day**	2997 (22.1)	2003 (43.7)	2818 (59.3)	3393 (52.0)	3869 (95.9)	<0.001

* *p*-trend values were calculated from a survey weighted linear regression modeling cholesterol intake quartiles as ordinal variable
